# Insurance Coverage Policies for Pharmacogenomic and Multi-Gene Testing for Cancer

**DOI:** 10.3390/jpm8020019

**Published:** 2018-05-16

**Authors:** Christine Y. Lu, Stephanie Loomer, Rachel Ceccarelli, Kathleen M. Mazor, James Sabin, Ellen Wright Clayton, Geoffrey S. Ginsburg, Ann Chen Wu

**Affiliations:** 1 Precision Medicine Translational Research (PROMoTeR) Center, Department of Population Medicine, Harvard Medical School and Harvard Pilgrim Health Care Institute, 401 Park Drive, Suite 401 East, Boston, MA 02215, USA; stephanie_loomer@harvardpilgrim.org (S.L.); ceccare1@msu.edu (R.C.); ann.wu@childrens.harvard.edu (A.C.W.); 2 Meyers Primary Care Institute, A Joint Endeavor of the University of Massachusetts Medical School, Reliant Medical Group and Fallon Health; 630 Plantation Street, Worcester, MA 01605, USA; Kathy.Mazor@meyersprimary.org; 3 Departments of Population Medicine and Psychiatry, Harvard Medical School, Boston, MA 02115, USA; jim_sabin@harvardpilgrim.org; 4 Center for Biomedical Ethics and Society, Vanderbilt University Medical Center, Nashville, TN 37232, USA; ellen.clayton@vanderbilt.edu; 5 Duke Center for Applied Genomics and Precision Medicine, Duke University Medical Center, Durham, NC 27710, USA; geoffrey.ginsburg@duke.edu

**Keywords:** pharmacogenomics, multi-gene testing, cancer, insurance coverage, tumor markers

## Abstract

Insurance coverage policies are a major determinant of patient access to genomic tests. The objective of this study was to examine differences in coverage policies for guideline-recommended pharmacogenomic tests that inform cancer treatment. We analyzed coverage policies from eight Medicare contractors and 10 private payers for 23 biomarkers (e.g., *HER2* and *EGFR*) and multi-gene tests. We extracted policy coverage and criteria, prior authorization requirements, and an evidence basis for coverage. We reviewed professional society guidelines and their recommendations for use of pharmacogenomic tests. Coverage for *KRAS*, *EGFR*, and *BRAF* tests were common across Medicare contractors and private payers, but few policies covered *PML/RARA*, *CD25*, or *G6PD*. Twelve payers cover at least one multi-gene test for nonsmall cell lung cancer, citing emerging clinical recommendations. Coverage policies for single and multi-gene tests for cancer treatments are relatively consistent among Medicare contractors despite the lack of national coverage determinations. In contrast, coverage for these tests varied across private payers. Patient access to tests is governed by prior authorization among eight private payers. Substantial variations in how payers address guideline-recommended pharmacogenomic tests and the common use of prior authorization underscore the need for additional studies of the effects of coverage variation on cancer care and patient outcomes.

## 1. Introduction

“Personalized medicine” is often described as providing “the right patient with the right drug at the right dose at the right time” [1]. Genomic tests are increasingly being used in clinical care to tailor treatment [2]. Approximately $5 billion was spent on genomic testing in 2010; expenditures are projected to reach $25 billion by 2021 [3]. As of 7 May 2018, there are over 54,538 tests for 11,169 conditions, 16,415 genes, and 506 laboratories according to the National Institutes of Health’s Genetic Testing Registry.

Genomic tests may identify individuals at risk of having a toxic response to a drug, thereby minimizing drug-related adverse events and associated costly consequences such as hospital admissions [4]. For example, pharmacogenomic tests for HLA-B*1502 can prevent potentially fatal carbamazepine-induced Stevens–Johnson syndrome. Pharmacogenomics tests can also identify individuals likely to respond to an intervention if molecular markers of therapeutic response are identified [5]. Examples include testing for epidermal growth factor receptor (*EGFR*) to determine the use of erlotinib in advanced nonsmall cell lung cancer (NSCLC), and *KRAS* testing to determine the use of cetuximab in advanced colorectal cancer. To aid clinical decision-making, the Centers for Disease Control and Prevention (CDC) has categorized genomic tests by level of evidence based on the Food and Drug Administration (FDA) labeling information, clinical practice guidelines, and systematic reviews [6]. This system defines about 50 genomic tests with sufficient evidence for clinical implementation (e.g., *KRAS* and human epidermal growth factor receptor 2 (*HER2*) tests to inform use of cetuximab in colorectal cancer and lapatinib in breast cancer, respectively).

At present, single gene tests cost about $200 whereas multi-gene panels are available for $600 [7]. Inter-related factors at the provider (e.g., specialty, training), patient (e.g., preferences), and health system (e.g., health insurance) levels affect patient access to genomic tests and related services [8]. Insurance coverage policies and levels of patient cost-sharing are major factors that influence who gets tested, screened, or treated, and when and which specific test is received [9,10,11,12]. To date, there has been a dearth of research on access to genomic tests. The objective of this study was to examine insurance coverage policies for guideline-recommended pharmacogenomic tests informing cancer treatment in order to identify potential access issues. We also sought to determine whether coverage policies are consistent with major clinical guidelines.

## 2. Methods

### 2.1. Pharmacogenomic and Multi-Gene Tests for Cancer

The CDC Office of Public Health Genomics (PHG) categorizes genomic tests by level of evidence based on the FDA labeling information, clinical practice guidelines, and systematic reviews [6,13]. This system defines ‘tier-1’ genomic tests as those that have a base of synthesized evidence that supports implementation in practice. We selected non-hereditary cancer-related pharmacogenomic tests for this study (Table 1). Because some of these tumor markers are now part of multi-gene tests, we also reviewed coverage policies for multi-gene panels that may govern access to tests for these biomarkers. The multi-gene panels we included are, however, not in the CDC’s tier-1 category at the time of the study. We did not include noncancer related multi-gene tests even if they were mentioned in some coverage policies as they are beyond the scope of this study. Whole genome sequencing and whole exome sequencing, which cover a large range of genes for noncancer indications, were not included in this study.

### 2.2. Guidelines Search and Data Extraction

We also reviewed publicly available guidelines from major professional societies and used them as a framework for examining coverage policies. The key evidence sources [6] that we included were: CDC’s PHG and Evaluation of Genomic Applications in Practice and Prevention (EGAPP), National Cancer Institute (NCI), National Comprehensive Cancer Network (NCCN), American Society of Clinical Oncology (ASCO), Center for Medical Technology Policy (CMTP), Blue Cross Blue Shield Association Technology Evaluation Center (BCBSA TEC has recently been replaced by Evidence Street), American College of Medical Genetics and Genomics (ACMG), Agency for Healthcare Research and Quality (AHRQ), and the United Kingdom’s National Institute for Health and Clinical Excellence (NICE). Search terms to identify relevant guidelines included: biomarker names, molecular profiling, multi-gene, and panel(s). We extracted the following information in August 2017 from the guidelines if the guideline recommended a pharmacogenomic test, the criteria, and the evidence basis for recommendation. Additionally, we extracted recommendations for multi-gene testing including suggested indications, for comparison with existing coverage policies mentioning multi-gene testing.

### 2.3. Medicare Contractors and Private Payers

Medicare is a public, nationwide health insurance program for people who are 65 and older in the US. We included all eight Medicare contractors: Novitas, National Government Services (NGS), Cigna Government Services (CGS), First Coast, Noridian, Palmetto, Wisconsin Physicians Service Insurance Corporation (WPSIC), and Cahaba. Private health insurance is often offered through an employer or organization but can also be purchased by an individual. We purposively selected the top US private payers based on the US News & World Report listing of largest third-party payers [15]: UnitedHealth, Blue Cross Blue Shield-Massachusetts (BCBSA-MA), Anthem, Aetna, Humana, Health Care Service Corporation (HCSC), Cigna, Highmark, and Independence Blue Cross (IBX). Together, these private payers cover care for about 182.3 million individuals across the US; membership estimates were derived from payer websites and Google searches. We also included Harvard Pilgrim Health Care (HPHC), a regional health plan serving the New England population; we included HPHC because this study is part of a larger project that includes a qualitative aim [16], which interviewed patients and providers from this regional health plan.

### 2.4. Policy Search and Data Extraction

For each payer, we searched for and reviewed coverage policies for 23 pharmacogenomic tests and cancer-related multi-gene tests (Table 1). Coverage policies were generally publicly accessible through the payer’s website, related websites (e.g., Centers for Medicare and Medicaid Services, CMS), and/or using search engines (Google). We searched for biomarker and drug names and key terms including genetic, gene, pharmacogenomics, biomarker, genomic, multi-gene, panel(s), sequencing, molecular profiling, and related Current Procedure Terminology (CPT) codes (e.g., 81275 for *KRAS*; three targeted sequencing CPT codes: 81445, 81450, and 81455). We reviewed and extracted the following features from identified documents: policy name and date; whether the policy covers the test; criteria for coverage e.g., medical condition; requirements for prior authorization; evidence basis for coverage; and CPT codes. Reimbursement of a genetic test subject to prior authorization is only permitted if a physician requests and obtains prior approval from the payer. Prior authorization is commonly used to manage utilization and costs for medical services and procedures and medications [17]. Our search for single gene tests was last updated in April 2017 and in December 2017 for multi-gene tests. We verified extracted information of coverage policies with six payers by sending them data extraction tables of publicly available policies to ensure accurate interpretation.

## 3. Results

### 3.1. Biomarker-Related Coverage Policies

Guidelines for single gene tests are generally in consensus; thus, we present our findings on the relevant coverage policies. Table 2 summarizes Medicare contractor and private payer coverage policies identified for pharmacogenomic tests guiding cancer treatments, including both the number of policies that cover the tests (one test may be included in multiple policies) and number of payers that cover the tests (regardless of how many policies included the test). Policies identified were dated from August 2015 until February 2017. We did not identify any Medicare national coverage determinations for study biomarkers. Across eight Medicare contractors, *KRAS*, *BRAF*, and *EGFR* were the most commonly covered single gene tests; six contractors covered testing for *KRAS*, while four contractors covered testing for *BRAF* and *EGFR*. Across 10 private payers, *KRAS*, *EGFR*, *BRAF*, and *ALK* were the most commonly covered single gene tests; nine payers covered testing for *KRAS*, *EGFR*, and *ALK*, and eight covered testing for *BRAF*.

There were differences across payers in terms of the presence of coverage policies for the specific pharmacogenomic tests included in the study, the content (e.g., covered indications), use of prior authorization requirements, and the evidence base cited (Table 2). Six private payers required prior authorization for single gene tests. The most common cited sources of references for coverage included: NCCN, ASCO, NCI, BCBSA TEC, and AHRQ. Evidence considered by payers for coverage decision making included randomized controlled trials and other study types, such as systematic reviews and cohort studies. The CPT codes specified by both Medicare and private payers were similar and included codes specific to biomarker testing and more general testing codes.

### 3.2. Multi-Gene Tests Related Guidelines

Few professional society guidelines were identified that specifically related to cancer multi-gene tests. Table 3 summarizes recent clinical recommendations for multi-gene testing. We identified substantial variation in the guidelines studied here. Evidence cited by these guidelines also differed such that no guidelines cited the same studies.

The guidelines that recommended multi-gene tests were NCCN [18], CMTP [19], ASCO [20], and ACMG [21], with NCCN and CMTP specifically recommending the tests for NSCLC. The CMTP also recommended such tests for other conditions including rare, uncommon, or stage IV solid tumors, e.g., lung and pancreatic cancers. These guidelines were largely based on small/medium sized observational studies, ranging from 31 to 2158 participants in each study [20,22,23,24,25,26,27,28,29,30,31,32,33,34,35,36,37]. Blue Cross Blue Shield Association TEC [38] and United Kingdom’s NICE [39] did not recommend multi-gene testing in clinical practice; both guidelines, however, were published in 2013, earlier than other guidelines. Blue Cross Blue Shield Association TEC cited four studies for five cancer indications, concluding that compelling evidence of clinical utility was not available for multi-gene testing and issues still exist which need attention, including varying approaches to the interpretation of multi-gene testing. NICE assessed multi-gene testing for detecting *EGFR* mutation in adults with locally advanced or metastatic NSCLC and concluded that there is insufficient data on clinical utility and cost-effectiveness [39]. All guidelines recognized the potential of multi-gene testing but also acknowledged the need for additional research on clinical utility of such tests in different conditions.

### 3.3. Multi-Gene Tests Related Coverage Policies

Table 4 summarizes coverage policies identified for cancer-related multi-gene tests. Policies identified were dated from April 2016 until December 2017. Eight Medicare contractors had 20 policies that mentioned and covered multi-gene tests. Specifically, six Medicare contractors covered multi-gene testing for NSCLC. Six of eight Medicare contractors also covered multi-gene tests for acute myelogenous leukemia (AML), myeloproliferative disease (MPD), and colorectal cancer (CRC). Palmetto’s policies were frequently cited by other Medicare contractors. We did not find prior authorization requirements for multi-gene tests by Medicare~contractors.

Six of ten private payers covered multi-gene testing for cancer in ten different policies. Six private payers specified coverage for NSCLC and two payers also covered multi-gene tests for myelodysplastic syndromes. In addition, Blue Cross Blue Shield provided coverage for many other indications including, but not limited to, colorectal cancer, gastrointestinal stromal tumors, and melanoma. IBX, HCSC, and Highmark, which all use eviCore, also provide coverage for colorectal cancer and melanoma. If a test was not covered, payers often cited limited clinical utility evidence as a justification and classified the test as “investigational/experimental.” Seven private payers required prior authorization for any multi-gene tests for cancer that is covered.

Overall, policies from both private payers and Medicare contractors that covered multi-gene tests were based on clinical guidelines as well as studies of different types (including clinical trials, cohort studies, clinical validation studies, expert opinions, editorials, and reviews such as GeneReviews). Across both public and private payers, multi-gene testing for cancer is most commonly covered for NSCLC, which is consistent with recommendations from NCCN, the predominant guideline for cancer. Payers varied in their coverage of multi-gene testing for other cancers, which reflects the fact that guidelines vary in their recommendations for the use of multi-gene tests in other cancers (Table 3).

## 4. Discussion

Genomic tests are increasingly used in clinical care and the associated businesses are booming. Insurance coverage policies can help govern the use of available resources for the population and support access to clinically valuable testing [40]. This study focused on insurance policies for guideline-recommended pharmacogenomic tests for cancer, either as single or multi-gene tests. We found substantial variations in how payers cover and manage pharmacogenomic testing for single gene tests as well as multi-gene tests, with more variations among private payers than Medicare contractors. Private payers commonly use prior authorization for these tests. Finally, we found discrepancies among coverage policies and clinical guidelines for multi-gene tests.

Variations in coverage policies among payers for single gene tests were present even though the pharmacogenomic tests included in the study were recommended by major guidelines for clinical implementation based on evidence [6]. It is important to note, however, lack of explicit policies do not necessarily translate to barriers to access for these single gene tests because Medicare contractors and private payers often do not have policies for specific genomic tests. Our interview study found that some payers do not view policies as necessary when clinical benefits are clear because policy development is time and resource intensive [41]. Perhaps this is not out of the ordinary given that many nongenetic medical services do not have specific policies beyond general statements about “medical necessity.”

Multi-gene testing is currently being used in clinical practice and could possibly be more widely used than single gene testing because of its potential to detect genomic alterations in hundreds of cancer-related genes. We found variations in coverage of multi-gene testing across private payers but relatively smaller variations among Medicare contractors. There are several possible explanations for observed variations. First, while two major guidelines recommend the use of multi-gene testing for NSCLC (including NCCN, the predominant guideline for cancer), one guideline does not recommend the use of multi-gene testing for NSCLC and two others do not specify an indication for testing. It is not surprising that six of 18 payers did not have policies for multi-gene tests for NSCLC. Coverage of multi-gene testing for other cancers is highly variable, which reflects the fact that guidelines vary in their recommendation for testing in other cancers. Second, there is limited evidence [17,38,42] on the clinical utility of multi-gene tests. Evidence cited by guidelines differs such that no guidelines cite the same studies, possibly due to the availability of published studies at the time of the assessment. The limited evidence and lack of clinical consensus prevent payers from judging whether a multi-gene test meets payers’ standards of medical necessity. The need for more research to generate evidence on clinical utility of multi-gene tests is widely recognized [17]. Third, payers’ consideration of different study types and interpretation of evidence could lead to differences in coverage. Few randomized controlled trials assessed the clinical utility of multi-gene tests given the complexities of such tests. Payers that cover multi-gene tests for NSCLC based their policies on clinical guidelines as well as a wide range of studies beyond randomized controlled trials including clinical validation studies, cohort studies, editorials, and reviews [43]. Finally, insurance coverage decisions are complex and they are based on evidence (including clinical utility evidence, and analytic and clinical validity information) as well as nonscientific factors (e.g., needs of the membership and lab contracting considerations), which are not outlined in coverage policies [41].

Importantly, we found that eight of ten private payers require prior authorization for all genomic testing, including single and multi-gene tests, to review medical necessity. Prior authorization generally obligates physicians to provide information on clinical indication and to specify gene(s) to be tested and patients to receive genetic counseling before and after genetic testing. Prior authorization is used to address the concern that, because multi-gene tests include a large number of genes, there is lack of transparency relating to the genes included in the panel tests [17,41,42]. Payers may determine the multi-gene test to be ‘investigational’ based on the combination of genes included that vary in their clinical utility. This concern relates to apprehensions about the numerous variants of unknown significance that might be reported to physicians. Such information may change physicians’ recommendation and influence patients’ decision to seek care that may not be appropriate. In order to grapple with the exponential growth in the types of tests and their growing use, and the limited expertise in this complex field [41], payers employ laboratory benefit management (LBM) companies to manage prior authorization applications and test utilization. This has occurred in other sectors of health care, for instance, pharmacy benefit management. The use of prior authorization is not limited to those payers who have contracted with an LBM. Of the eight private payers that require prior authorization for single gene and/or multi-gene testing, two do not use an LBM.

Variations in coverage policies and prior authorization requirements among payers create confusion for patients and clinicians as to who has access to these tests and how they are used in clinical practice [16]. We have several recommendations to reduce confusion and coverage variations. First, understanding of payer coverage needs to be improved for genomic tests through ongoing, systematic analyses of coverage policies and structured reporting. Coverage policies have a significant role in determining patient access to genomic tests but, as demonstrated by our study, they are extremely complex. Payers present information differently in their policies; for instance, some include a range of genomic tests in the same policy and some may require use of specific test products (e.g., Cologuard, Therascreen for *KRAS*). Registries of coverage policies, such as our own registry and the TRANSPERS Payer Coverage Policy Registry [17], can provide an objective, structured tool for research to examine coverage policies across payers, diseases or tests. These studies could facilitate greater transparency in payer coverage decision-making, identify evidence gaps, and enhance patient access to proven genomic tests. In addition to coverage policies, our registry also captures valuable information on prior authorization and major clinical guidelines. While prior authorization can help ensure appropriate use of genomic tests, it can also affect access to tests. Furthermore, it is important to examine whether coverage policies are consistent with clinical guidelines. Maintaining and keeping this type of registry up-to-date is a major undertaking, as the policies, prior authorization requirements, and guidelines are updated as evidence accumulates and/or as demand from patients and clinicians for access to genomic tests increases. Second, a single agency with appropriate expertise to assess evidence for coverage decisions, such as Hayes Inc., could reduce variations in coverage across payers. For instance, coverage for cancer-related multi-gene testing by Medicare contractors is relatively consistent because other Medicare contractors often adopt Palmetto’s assessments of genomic tests, whereas substantial variations exist among private payers as they make their coverage decisions and policies independently. In addition, the use of LBM companies by payers is rapidly expanding to meet the need for systemization of evidentiary review and automate updates to keep up with the deluge of data in the literature [41]. Finally, increased dialogue and sharing of information among payers could also create a more harmonious and uniform system.

The strength of our study is that it is one of the first to systematically analyze the coverage of guideline-recommended pharmacogenomic tests for cancer, either as single or multi-gene tests, as well as discrepancies among coverage policies and clinical guidelines. Nevertheless, this study has several limitations. First, we included all Medicare contractors but not state Medicaid programs (Medicaid programs provide health insurance to low-income citizens across the US). We included only 10 private payers; however, we deliberately selected the top private payers across the US that provide coverage for about 182.3 million individuals. We also included major guidelines available publicly; however, there are other sources used by payers that we do not subscribe to and are therefore not included in the study (e.g., Hayes Inc., Lansdale, PA, USA). Second, we focused on tumor markers for guiding cancer treatments and those that were classified by CDC as tier-1 category. We did not attempt to examine insurance coverage for multi-gene tests unrelated to these tumor makers; thus, access to many multi-gene tests was beyond the scope of this study (e.g., multi-gene tests for Lynch Syndrome) [17]. Third, our analysis of published coverage policies did not aim to describe comprehensively the complexity of coverage decision making. There are inherent limitations of published coverage policies as these documents do not discuss all the factors that determine coverage, including judgment in the interpretation of evidence. Fourth, the FDA recently approved a next generation sequencing-based in vitro diagnostic test that can detect genetic mutations in 324 genes and two genomic signatures in solid tumors [44]. The CMS also finalized a national coverage determination for this next generation sequencing-based diagnostic test [45]. The FDA’s approval and CMS coverage for this multi-gene test is a big step forward in genomic health care and could influence future coverage and utilization of multi-gene tests (for cancer and beyond). Finally, coverage policies do not directly translate to reimbursement (e.g., whether prior authorization for the individual patient is approved).

## 5. Conclusions

Genomic tests are increasingly used in clinical care and the associated businesses are booming. Insurance coverage policies can help govern the use of available resources for the population and support access to clinically valuable testing. Our findings of variations in how payers are addressing pharmacogenomic tests and the common use of prior authorization underscore the need for additional research into the impact of these variations on cancer care and outcomes. Ongoing systematic analyses of coverage policies and prior authorization requirements considering key clinical recommendations are needed to improve understanding of access to and use of genomic tests.

## Figures and Tables

**Table 1:** Study drugs and pharmacogenomic and multi-gene tests.

Biomarker	Drug Name	Condition	FDA Drug Approval Date
*KRAS*	Cetuximab	Metastatic colorectal cancer	2/12/2004
	Panitumumab	Metastatic colorectal cancer	9/27/2006
*HER2*	Trastuzumab	Gastroesophageal junction adenocarcinoma	9/25/1998
	Trastuzumab	Invasive breast cancer	9/25/1998
	Pertuzumab	Invasive breast cancer	6/8/2012
	Ado-trastuzumab emtansine	Metastatic breast cancer	2/22/2013
	Lapatinib	Advanced or metastatic breast cancer	3/13/2007
*BRAF*	Trametinib	Unresectable or metastatic melanoma	5/29/2013
	Dabrafenib	Unresectable or metastatic melanoma	5/29/2013
	Vemurafenib	Unresectable or metastatic melanoma	8/17/2011
*EGFR*	Afatinib	Metastatic Nonsmall cell lung cancer	7/12/2013
	Erlotinib	Metastatic Nonsmall cell lung cancer	11/18/2004
*ALK*	Crizotinib	Nonsmall cell lung cancer	8/26/2011
*BCR-ABL1*	Dasatinib	Chronic myeloid leukemia/Acute lymphoblastic leukemia	6/28/2006
	Imatinib	Chronic myeloid leukemia/Acute lymphoblastic leukemia	5/10/2001
	Bosutinib	Chronic Myeloid Leukemia	9/4/2012
	Nilotinib	Chronic Myeloid Leukemia	10/29/2007
*c-Kit protein*	Imatinib	Gastrointestinal stromal tumors	5/10/2001
*PDGFR*	Imatinib	Myelodysplastic/Myeloproliferative diseases	5/10/2001
*CD20*	Tositumomab	Non-Hodgkin’s lymphoma	6/27/2003
*G6PD*	Rasburicase	Leukemia, lymphoma	7/12/2002
*CD25*	Denileukin diftitox	Cutaneous T-cell lymphoma	2/05/2009
*PML/RARA*	Arsenic trioxide	Acute promyelocytic leukemia	9/25/2000
**Cancer Related Multi-Gene Tests**			CPT Code *
Targeted genomic sequence analysis of 5–50 genes for solid tumors (e.g., *ALK, BRAF, CDKN2A, EGFR, ERBB2, KIT, KRAS, NRAS, MET, PDGFRA, PDGFRB, PGR, PIK3CA, PTEN,* and *RET*)			81445
Targeted genomic sequence analysis of 5–50 genes for hematologic malignancies (e.g., *BRAF, CEBPA, DNMT3A, EZH2, FLT3, IDH1, IDH2, JAK2, KRAS, KIT, MLL, NRAS, NPM1,* and *NOTCH1*)			81450
Targeted genomic sequence analysis of 51 or greater genes for solid tumors or hematologic malignancies (e.g., *ALK*, *BRAF*, *CDKN2A*, *CEBPA*, *DNMT3A*, *EGFR*, *ERBB2*, *EZH2*, *FLT3*, *IDH1*, *IDH2*, *JAK2*, *KIT*, *KRAS*, *MLL*, *NPM1*, *NRAS*, *MET*, *NOTCH1*, *PDGFRA*, *PDGFRB*, *PGR*, *PIK3CA*, *PTEN*, and *RET*)			81455

* All Current Procedural Terminology (CPT) codes come from the American Medical Association [14]. FDA-Food and Drug Administration.

**Table 2:** Coverage policies for pharmacogenomic tests guiding cancer treatments by Medicare contractors (*n* = 8) and private payers (*n* = 10).

	Number of Policies that Mention & Cover the Test		Number of Insurers that Cover the Test		Covered Conditions		Key References Cited in Coverage Policies		CPT Codes Specified in Policies	
	Medicare	Private	Medicare	Private	Medicare	Private	Medicare	Private	Medicare	Private
*KRAS*	6	12	6	9	mCRC ^3^ NSCLC CML ALL	mCRC ^3^ Adeno- carcinoma NSCLC	EGAPP; FDA; NCCN; ASCO; AHRQ; AMA; USPSTF; CDC; Palmetto	BCBSA TEC; EGAPP; ASCO; NCCN; ACG; CAP TEC	81275-276 81311 81405-406	81275-276 ^2^ 81405
*HER2*	1	6	1	6	Breast cancer ^3^ Gastric adenocarcinoma	Breast cancer ^3^ Gastric adenocarcinoma NSCLC	NCCN; ASCO; CDC; AHRQ; AMA; USPSTF	Hayes; NCCN; NICE; ECRI; ASCO; BCBSA TEC; USPSTF; ACMG; AHRQ	None	83950 88360-361 ^1^
*BRAF*	4	11	4	8	Melanoma ^3^ NSCLC Hairy cell leukemia CRC Brain cancer Thyroid cancer Ovarian/ uterine cancer	Melanoma ^3^ NSCLC GISTs mCRC Hairy cell leukemia Lynch syndrome	EGAPP; FDA; NCCN; ASCO; AHRQ; AMA; USPSTF; CDC; Palmetto	FDA; BCBSA TEC; NCCN; AHRQ; EGAPP	81210	81210 ^2^ 88363 81406
*EGFR*	4	12	4	9	NSCLC ^3^ Brain cancer	NSCLC ^3^ CNS cancers	EGAPP; FDA; NCCN; ASCO; AHRQ; AMA; USPSTF; CDC; Palmetto	ASCO; CAP; NCCN; FDA; CAP/IASLC/AMP	81235	81235 ^2^
*ALK*	2	10	2	9	NSCLC ^3^	NSCLC ^3^ Lymphoma IMT	EGAPP; FDA; NCCN; ASCO; AHRQ; AMA; USPSTF; CDC; Palmetto	NCCN; NIH; ASCO	None	81401 ^1^ 81479 88271 88274 88291 88367-368
*BCR-ABL1*	3	8	3	8	CML ^3^ ALL ^3^ CMML	CML ^3^ ALL ^3^	EGAPP; FDA; NCCN; ASCO; AHRQ; AMA; USPSTF; CDC; Palmetto	WHO; FDA; NCCN; AHRQ; NCI; ACS; AMP; Hayes	81206-208 81219 81270 81402-403	88170 88275 81206-208 ^2^ 81401 88271
*c-Kit*	2	3	2	3	GISTs ^3^ Melanoma AML ALL MDS	GISTs ^3^ Melanoma AML ALL MDS	EGAPP; FDA; NCCN; ASCO; AHRQ; AMA; USPSTF; CDC; Palmetto	NCCN	81272-273	81272-273 ^1^ 88184
*PDGFR*	1	1	1	1	MDS/MPN ^3^ GISTs	MDS/MPN ^3^ GISTs	AHRQ; AMA; CDC; USPSTF	None	None	81314 81404
*CD20*	0	2	0	2	N/A	NHL ^3^	AMA; Palmetto	None	None	88184
*G6PD*	2	0	2	0	None specified	N/A	AHRQ; CDC; USPSTF	N/A	81247-249	N/A
*CD25*	0	0	0	0	N/A	N/A	N/A	N/A	N/A	N/A
*PML/RARA*	2	3	2	2	Acute promyelocytic leukemia	Acute promyelocytic leukemia	AHRQ; CDC; USPSTF	NCCN; FDA	81315-316	81315-316

^1^ Most commonly used CPT code for biomarker testing; ^2^ Specific CPT code for biomarker testing; ^3^ Indicates FDA approved indication. All coverage counts based on medical coverage policies: last updated on 04/2017 for single gene tests; policies identified were dated from 08/2015 until 02/2017. Medicare contractors include Novitas, Noridian, Palmetto, Cahaba, Wisconsin Physicians Service Insurance Corporation (WPSIC), National Government Services (NGS), Cigna Government Services (CGS), and First Coast. Private payers include Harvard Pilgrim Health Care, United Healthcare, Anthem, Aetna, Cigna, Independence Blue Cross, Humana, Highmark, HCSC, and Blue Cross Blue Shield of Massachusetts. Abbreviations: CPT-Current Procedural Terminology. Conditions: ALL-acute lymphoblastic leukemia, AML-acute myeloid leukemia, CML-chronic myeloid leukemia, GISTs-gastrointestinal stromal tumors, IMT-inflammatory myofibroblastic tumor, MDS/MPN-myelodysplastic/myeloproliferative diseases, mCRC-metastatic colorectal cancer, NHL-Non-Hodgkin’s lymphoma, and NSCLC-nonsmall cell lung cancer. References: ACG-American College of Gastroenterology, ACMG-American College of Medical Genetics and Genomics, ACS-American Cancer Society, AHRQ-Agency for Healthcare Research and Quality, AMA-American Medical Association, AMP-Association for Molecular Pathology, ASCO-American Society of Clinical Oncology, BCBSA TEC-Blue Cross Blue Shield Association Technology Evaluation Center, CAP TEC- College of American Pathologists Technology Evaluation Center, CDC-Centers for Disease Control and Prevention, ECRI-Emergency Care Research Institute, EGAPP-Evaluation of Genomic Applications in Practice and Prevention, IASLC-International Association for the Study of Lung Cancer, NCCN-National Comprehensive Cancer Network, NCI-National Cancer Institute, NICE-National Institute for Health and Care Excellence, USPSTF-United States Preventive Services Task Force, and WHO-World Health Organization.

**Table 3:** Multi-gene testing recommendations by Professional Society Guidelines.

	Society, Published Year	Indications Mentioned	Criteria/Reasoning	References Cited
**Recommend Multi-gene Testing**	ACMG, 2013	None specified	Targeted multi-gene testing is recommended for genetically heterogeneous disorders and oncology applications. By limiting the content of the test to just the regions relevant to a given disease, the resulting data usually have higher analytical sensitivity and specificity for detecting mutations.	NIH, College of American Pathologists
	ASCO, 2015	None specified	Testing option recommendations: Multi-gene testing may be efficient in circumstances that require evaluation of multiple high-penetrance genes of established clinical utility Panel testing may identify mutations in genes associated with cancer risks and mutations in high-penetrance genes Somatic mutation profiling can identify driver mutations in the cancer that could serve as treatment targets Tests being done to guide cancer treatment are often time sensitive and should only include those tests that guide therapy. Management of individuals/families with mutations in moderately penetrant genes must include: A review of the literature so that family can determine the most appropriate screening/prevention plan Patients to have proper counseling, as the results of panel testing may not always be straight-forward Challenges, risks, and benefits explained to patients prior to testing Patient preferences should be included in decision making regarding the most appropriate test	NIH, Cigna, observational study (*n* = 194 and 586), cross-sectional study (*n* = 2158), online surveys (*n* = 225)
	CMTP, 2015	NSCLC, advanced stage solid tumors, hematologic malignancies	Testing of 5 or more genes is recommended with the following criteria: Must be clinically relevant as cited by NCCN or ASCO; OR be cited in the label of an FDA-approved companion diagnostic; AND is not more expensive than the cost of individual testing Testing of 50 or more genes is recommended with the following criteria/indications: NSCLC Rare, uncommon, or stage IV solid tumors, e.g., lung and pancreatic cancers Exhausted other treatment options (including unresponsiveness of treatments)	NCCN, Palmetto, review article
	NCCN, 2017	NSCLC	Broader molecular profiling with the goal of identifying rare driver mutations for which effective drugs may already be available, or to appropriately counsel patients regarding availability of clinical trials. Broad molecular profiling is a key component of the improvement of care of patients with NSCLC.	ASCO, cohort study (*n* = 31), retrospective observational study (*n* = 419)
**Do Not Recommend Multi-gene Testing**	BCBSA TEC, 2013	None specified	Limited studies, poor study designs, plus a number of practical issues pertinent to application of molecular marker profiling have not been sufficiently resolved for clinical implementation. For example, the relative accuracy and precision of different DNA sequencing methods are under investigation and may exhibit variability secondary to training and experience of laboratories and personnel. Optimal informatics methods to handle large amounts of sequencing data and reconstruct them into clinically actionable information displays remain problematic.	Two clinical trials (*n* = 68 and 86), observational study (*n* = 460), methods study
	NICE, 2013	NSCLC	For non-Sanger sequencing based tests and for tests such as Therascreen *EGFR* Pyro Kit and next generation sequencing, there is insufficient evidence and therefore, no recommendations can be made on their use.	NCCN, systematic review and cost-effective analysis

Years reflect most recent, available published guidelines mentioning or specific to molecular profiling/multi-gene testing.

**Table 4:** Coverage policies for cancer related multi-gene tests by Medicare contractors (*n* = 8) and private payers (*n* = 10).

	Medicare	Private
Policy mentions & covers at least one multi-gene test	20	10
Policy mentions & does not cover any multi-gene tests or covers only medically necessary genes within the panel	3	6
Payers require prior authorization for testing	N/A	7
Payers that cover at least one multi-gene test for NSCLC	6	6
Payers that cover at least one multi-gene test for conditions other than NSCLC	6	5
Covered conditions	Acute myelogenous leukemia CRC MPD NSCLC	MPD Thryoid cancer NSCLC Others ^1^
Key references cited	FDA, NCCN, WHO, Palmetto	BCBSA TEC, ECRI, FoundationOne Foundation Medicine TA, Hayes Inc, NCCN, NICE, SGO, NCI, FDA, ACOG, USPSTF, ACS, ANZHSN, ASCO, ACMG

All coverage counts based on medical coverage policies; last updated on 10/2017 for multi-gene tests; policies identified were dated from 01/2016 until 02/2017; Medicare contractors include First Coast, Cahaba, CGS, National Government Services, Noridian, Novitas, Palmetto, and Wisconsin Physicians Services Insurance Company; Private payers include Aetna, Blue Cross Blue Shield of Massachusetts (BCBS), Anthem, Cigna, Harvard Pilgrim Health Care, HCSC, Highmark, Humana, Independence Blue Cross, and United Healthcare. ^1^ BCBS covers many indications including: Solid Tumor NGS Panel: B-Cell NHL, bladder urothelial carcinoma, breast cancer, cholangiocarcinoma, endometrial carcinoma, GI stromal tumor, glioma, medulloblastoma, melanoma, meningioma, neuroblastoma, rare tumors, stomach/esophageal cancer, T-cell NHL, acute myeloid leukemia, B-ALL, B-cell NHL, myelodysplasia, myeloproliferative diseases, T-ALL, and T-Cell NHL. Abbreviations: ACOG-American College of Obstetricians and Gynecologists; ACS-American Cancer Society; ANZHSN-Australia and New Zealand Horizon Scanning Network; CRC-colorectal cancer; MPD-myeloproliferative disease; SGO-Society of Gynecologic Oncology.
